# Nutritional Status and Basic Hygiene Practices of Rural School Age Children of Savar Region, Dhaka, Bangladesh

**DOI:** 10.5195/cajgh.2018.282

**Published:** 2018-03-14

**Authors:** Sahadat Hossain, Fahad Ahmed, Shakhaoat Hossain, Tajuddin Sikder

**Affiliations:** 1 Department of Public Health and Informatics, Jahangirnagar University, Dhaka, Bangladesh; 2 Department of Environmental Sciences, Jahangirnagar University, Dhaka, Bangladesh

**Keywords:** Children, Nutritional status, Hygiene, Body Mass Index, Bangladesh

## Abstract

**Introduction:**

School children in the developing countries are susceptible to nutrition related health problems due to demographic and socio-economic factors, as well as limited access to food. Since BMI is a common proxy measure used to evaluate nutritional status, the aim of this study was to investigate the BMI categories in school-aged children in Dhaka, Bangladesh.

**Methods:**

A cross sectional study of 155 children, aged 6–12 years was conducted at Jahangirnagar University School in Dhaka, Bangladesh. The data collection was performed by in-person interviews and semi-structured questionnaires. Descriptive statistics, χ2 test, Fisher’s exact test, and One-Way ANOVA test were performed to compare the variables based on BMI percentiles. Data were analyzed using the Microsoft Excel program (version 2010).

**Results:**

Mean BMI of the students was 17.27 (SD=3.16). The prevalence of underweight and overweight/obesity was 11.0% and 25.8%, respectively. Categories of BMI percentiles were associated with birth order (p=0.026), personal hygiene practices (washing hands after coming home from outside (p<0.001) and before meal (p=0.045)), brushing teeth (p<0.001), the number of food items consumed daily (p<0.001), and mothers’ occupation (p=0.006). In context of basic hygiene practice, 61.3 % of respondents washed hands after coming home from outside, and 93.5 % reported washing hands before the meals.

**Conclusions:**

This study revealed that more than one third of the students had abnormal BMI. BMI screening in rural schools needs to be recommended in early grades for all children.

The school age (6–12 years old) is a dynamic period of growth and development,^1^ with rapid changes in physical, mental, and social skills.^2^ In the developing countries, the burden of morbidity and mortality associated with malnutrition and infectious diseases among the school age children is growing.^3^ Undernutrition still remains a problem in school age children in developing countries, and is associated with stunted growth, lower body weights, and shorter heights.^4^, ^5^ A large proportion of children in these countries is impacted by anemia,^6^ vitamin A deficiency,^7^ and parasitic infections^8^, which negatively impact their nutritional status,^8^ cognitive development, and school performance.^9^,^10^

Though multiple factors are associated with malnutrition, poor hygiene practice is one of the major factors linked to malnutrition among the school age children.^11^, ^12^ Nutritional status of children in many developing countries is affected by hygiene status, such as lack of clean water, poor sanitation, poor hygiene practices, and lack of access to toilets.^13^ This is an especially important issue for children under the age of five, as water- and sanitation-related diseases are the leading causes of early morbidity and mortality.^13^

Bangladesh, a densely populated developing country in South Asia, has experienced rapid demographic and epidemiological transition over the past few decades. It is facing paradoxical health challenges related to high prevalence of both underweight and overweight problems.^14^ According to the 2014 Bangladesh Health and Demographic Survey, 33% of children in the country were underweight.^15^ On the other hand, a recent countrywide epidemiological study reported that among 6–15 years old children 9.5% were overweight and 3.5% were obese.^16^

According to the World Health Organization (WHO), childhood obesity is a universal problem gradually affecting a large number of low-and middle-income countries.^17^ The prevalence of childhood obesity is approaching an epidemic proportion in many economically developed countries such USA, Canada, Australia, and several European countries.^18^ However, its prevalence in developing countries oftentimes reaches epidemic proportions faster than in the developed world.^19^ It has been reported that overweight and obesity in childhood is associated with increased risk of both, premature mortality and morbidity in adults, particularly due to cardiometabolic factors.^20^ These studies suggest that even in a resource-poor setting like Bangladesh, where under-nutrition is still prevalent, overweight and obesity could emerge as a major public health challenge in the near future.

Lack of information concerning nutrition and hygiene practices among school children in Bangladesh increases the complexity of providing appropriate public heath intervention programs. Although several studies on hygiene practices were performed with different population groups in Bangladesh,^21^, ^22^ to our knowledge, none of the existing studies focused on the school aged children. Thus, due to the limited published data on the BMI status and basic hygiene practices of school aged children in rural Bangladesh, the goal of this study was to assess BMI status and basic hygiene practices in school age children studying at Jahangirnagar University School.

## Methods

### Study population

Before the data collection, investigators obtained school management’s permission to approach prospective study participants. The school management notified students and their parents or guardians about the objectives of the study. At the time of data collection, we also took verbal consent from the respondents.

In the initial recruitment step, the school’s management provided the research team with the list of enrolled students. Prospective participants were selected from this list based on the following inclusion criteria: age group of 6–12 years, record of being present in the school for at least 90% of the school days, and interest in participating. After creating a list of students that met the inclusion criteria, we obtained a simple random sample of the respondents who were later asked to participate in the interview.

School children (155 participants, 90 males and 65 females) from families with varied levels of income (high, middle, low, and extremely low) were enrolled into a cross-sectional survey study by using random sampling method in May–June of 2015 at Jahangirnagar University School, Savar region, Dhaka, Bangladesh.

### Measurements

A pre-designed and pre-tested semi-structured questionnaire was used to interview the participants to collect information on socio-demographic characteristics, hygiene practices, and the number of food items consumed on the daily basis. The semi-structured questionnaire was pre-tested with 25 children of comparable age and socioeconomic characteristics from a neighboring school. Necessary modifications were made to the questionnaire before the start of the data collection. To better understand the socio-demographic characteristics of the respondents, information related to their age, sex, birth order, and parents’ occupation was collected. Hygiene practices were assessed with the questions focusing on washing hands before a meal, after coming home from outside, and after using the toilet. For washing hands, there was a question assessing if soap or detergent was used for hand washing. In order to assess oral hygiene, respondents were asked how many times they brush their teeth in a typical day. To assess the dietary history, students were asked how many meals they typically consumed in a typical day. They were also asked to comment on the types of foods they consumed in a typical day, and how many food items their meals included.

By following standard procedures, anthropometric measurements (height and weight) were collected from all participants by trained field workers.^23^ The ZZJKH-01 scale was used to measure the height and weight of the participants. At the time of measurement, the students were dressed in light clothing and their shoes were removed. To calculate Body Mass Index (kg/m^2^), weight was measured in kilograms and divided by the squared height in meters.^24^ Participants with a BMI < 5^th^ percentile were classified as underweight, those with a BMI 5th to < 85th percentile was considered as healthy weight, BMI 85th to < 95th percentile were classified as overweight, and the BMI ≥ 95th percentile was categorized as obese.^25^

### Statistical Analysis

Descriptive statistics were used to analyze the study results. Chi-square test and Fisher’s exact test were used to identify the significant relationship between categorical variables, including demographic (age, sex birth order, and parents’ occupation), anthropometric (BMI categories), nutrition, and hygiene. BMI was compared based on gender, parents’ occupation, and frequency of daily food intake categories using one-way ANOVA test. Data were processed and analyzed using the Microsoft Excel program (version 2010). All the p values were two-tailed and statistical significance was set at the conventional cut-off of p ≤ 0.05.

## Results

A total of 155 children were interviewed (Table 1) of which 90 were male (58.1%) and 65 were female (41.9%), 81 (52.3%) were aged 6–9 and 74 (47.7 %) were aged 10–12. Almost 82% of them were from the nuclear families with four or less members in the household, and 43.2% of the children were in the second birth order. We found that 16% of mothers were employed outside of their households, whereas about 50% of fathers were involved in service jobs.

The prevalence of underweight, healthy weight, and overweight/obese children was 11.0%, 63.2%, and 25.8% respectively (Table 2). More than one-third of the students deviated from the healthy weight category. Female students were underweight (64.7%) more often than males, whereas male students were overweight/obese (55.0%) more often than females. The study showed that birth order was significantly associated with the categories of BMI percentile (p-value=0.026; 95% CI: 0.001 – 0.051). Mother’s occupation was also a significant factor in determining the health status of the study students (χ^2^(2, N=155)=11.17, p=0.006). We found that almost 93% of students reported washing their hands before the meals. Similarly, about 98% students reported washing hands with soap after using toilet. In addition, the rate of hand washing after coming home from outside was relatively low (61%). Majority of the students (53.1%; χ^2^(2, N=155)=27.75, p<0.001) who were in the healthy weight category did not wash or irregularly washed hands after coming home from outside. The study revealed that 90.3% of students reported brushing their teeth once per day, and 9.7% reported brushing their teeth twice per day.

Table 3 shows that more than half of the students (56%) consumed less than three food items per day, and their average BMI was 15.30 (SD=1.59). On the other hand, 22.6% of students who consumed more than four items of foods daily had on average BMI of 21.08 (SD=2.84).

Table 4 summarizes the association of daily food intake frequency with other variables. Daily food intake frequency was significantly different between age groups, χ^2^(2, N=155)= 7.34, p=0.026, and categories of BMI percentile, χ^2^(4, N=155)= 76.08, p<0.001. The study showed that 74.3% of overweight/obese students consumed foods more than four times per day. No cases of underweight were reported among students who consumed food three or more times daily. All of the respondents reported that they ate rice regularly. Rice, fish, pulses, and beans (such as dry pea and lentils) were the most commonly consumed foods among the respondents who reported consuming two food items daily. Very few children reported consuming vegetables and fruits in their daily meals. We also found that there is a significant relationship between daily food intake frequency and the dental hygiene, χ^2^(2, N=155)= 17.11, p<0.001.

## Discussion

The key finding of this study is that more than one third of the students deviated from healthy body weight. Age, sex, birth order, mother’s occupation, hand hygiene, and total daily number of meals were significantly associated with BMI level. Female students were found to be underweight more often than male students, while male students were more often found to be overweight/obese. The findings of this study showed that the number of underweight students was six percent lower and the number of overweight/obese students was twice as high when compared to the recent large scale study conducted in Bangladesh.^16^ Additionally, the number of underweight in this study was lower than that reported in the countrywide epidemiological study, and the overall number of overweight/obese students was almost doubled compared to what was previously reported.^26^ These findings warrant additional investigation.

In this study, it was found that more than half of the respondents (56%) who consumed foods less than three times per day had the average BMI of 15.30 kg/m^2^ (SD=1.59), which has important implication for children’s nutritional status. Access to food is hypothesized to be the primary reason behind low food intake frequency. Published evidence suggests that food unavailability is associated with poor health status, lower cognitive and academic attainment, and psychosocial problems among school age children.^27^ A study in Malaysia showed that children in food-insecure households were 2.15 times more likely to be underweight.^28^ From previously published reports on food consumption patterns in Bangladesh, we found that the main sources of carbohydrate were rice and wheat, while fish, poultry, eggs, and pulses were the main sources of protein.^29^, ^30^ The overall personal hygiene practices reported in this study were similar to the previous studies among different population groups in the country.^22^, ^31^, ^32^

A major strength of this study is that it followed the standard operating procedures with uniform methodology for data collection from all participants. The other strength is that participants of this study came from heterogeneous family background because the school consisted of high, middle, low, and extremely low family income children. Thus, our findings are generalizable to multiple population groups.

This study has some limitations, including study implementation in small geographic area, insufficient data on dietary patterns of the local populations, and lack of ability to compare these data to the populations outside this rural area. In addition, hygiene practices could have been over reported, as it is common with all self-reported practices in personal interviews.

Nutritional status of school age children should be closely monitored in order to improve their physical and mental health. Parents from all income categories (high, middle, low, and extremely low family income) should be trained to maintain healthy diet irrespective of their financial status, such as increasing consumption of plant proteins, dietary fiber, fresh fruits, and vegetables. In this study, very few children reported consuming vegetables and fruits in their daily meals, suggesting that most students do not fulfill their nutritional needs by current food consumption patterns.

This study demonstrates that although basic self-reported hygiene practice of rural school age children is satisfactory and the prevalence of underweight is in relatively low, the prevalence of overweight/obesity is relatively high in comparison with previously reported countrywide statistics. The overweight and/or obesity of school aged children can be an emerging public health problem in Bangladesh. This represents a unique public health challenge that will need to be addressed with future targeted public health interventions. Nutrition programs and active lifestyle health policies should be focused on school-aged children, especially in rural Bangladesh. Health monitoring study for the school aged children would be further conducted to better understand behavioral patterns of these children along with the general perception of obesity. This study can be viewed as a baseline work for other researchers focusing on childhood nutrition in Bangladesh and similar countries.

## Figures and Tables

**Table 1:** Socio-demographic characteristics of the participants

	Total (%l)	Male (%)	Female (%)
Age Group			
6 – 9	81 (52.3)	46 (51.1)	35 (53.8)
10 – 12	74 (47.7)	44 (48.9)	30 (46.2)
Birth Order			
1^st^	40 (25.8)	22 (24.4)	18 (27.7)
2^nd^	67 (43.2)	38 (42.2)	29 (44.6)
>2^nd^	48 (31.0)	30 (33.3)	18 (27.7)
Family Structure			
Small (≤ 4 Members)	127 (81.9)	75 (83.3)	52 (80)
Large (> 4 Members)	28 (18.1)	15 (16.7)	13 (20)
Father’s Occupation			
Service	77 (49.7)	45 (50)	32 (49.2)
Unemployed/Business/Other	78 (50.3)	45 (50)	33 (50.8)
Mother’s Occupation			
Service/Other	25 (16.1)	75 (83.3)	55 (84.6)
Homemaker	130 (83.9)	15 (16.7)	10 (15.4)

Total	155	90	65

**Table 2:** Distribution of socio-demographic characteristics and hygiene practices by the categories of BMI percentile

	Total (%)	Underweight (%)	Healthy weight (%)	Overweight/Obese (%)	P value	95% CI
Age group						
6 – 9	81 (52.3)	6 (35.3)	51 (52.0)	24 (60.0)	0.213	0.148 – 0.277
10 – 12	74 (47.7)	11 (64.7)	47 (48.0)	16 (40.0)		
Sex						
Male	90 (58.1)	6 (35.3)	62 (63.3)	22 (55.0)	0.077	0.035 – 0.119
Female	65 (41.9)	11 (64.7)	36 (36.7)	18 (45.0)		
Birth order						
1^st^	40 (25.8)	5 (29.4)	17 (17.3)	18 (45.0)	0.026^a^	0.001 – 0.051
2^nd^	67 (43.2)	8 (47.1)	47 (48.0)	12 (30.0)		
>2^nd^	48 (31.0)	4 (23.5)	34 (34.7)	10 (25.0)		
Father’s occupation						
Service	77 (49.7)	9 (52.9)	47 (48.0)	21 (52.5)	0.845	0.788 – 0.902
Unemployed/Business/Other	78 (50.3)	8 (47.1)	51 (52.0)	19 (47.5)		
Mother’s occupation						
Service/Others	25 (16.1)	6 (35.3)	18 (18.4)	1 (2.5)	0.006^a^	0.000 – 0.019
Homemaker	130 (83.9)	11 (64.7)	80 (81.6)	39 (97.5)		
Washing hand after coming home from outside						
Yes	95 (61.3)	11 (64.7)	46 (46.9)	38 (95.0)	<0.001	0.000 – 0.019
No/Irregular	60 (38.7)	6 (35.3)	52 (53.1)	2 (5.0)		
Washing hand before meal						
Yes	145 (93.5)	17 (100)	88 (89.8)	40 (100)	0.045^a^	0.012 – 0.078
No/Irregular	10 (6.5)	-	10 (10.2)	-		
Washing hand after toilet with soap						
Yes	152 (98.1)	17 (100)	95 (96.9)	40 (100)	0.677^a^	0.604 – 0.751
No/Irregular	3 (1.9)	-	3 (3.1)	-		
Brushing teeth per day						
One time	140 (90.3)	17 (100)	97 (99.0)	26 (65.0)	<0.001^a^	0.000 – 0.019
Two times	15 (9.7)	-	1 (1.0)	14 (35.0)		

Total	155 (100)	17 (11.0)	98 (63.2)	40 (25.8)		

a Fisher’s Exact Test

**Table 3:** Comparison of mean BMI between categories of socio-demographic characteristics and daily food intake

			95% Confidence interval for mean		
	N	Mean (±SD)	Lower bound	Upper bound	P value^a^
Gender					
Male	90	17.63 (3.25)	16.95	18.31	0.092
Female	65	16.76 (2.97)	16.03	17.50	
Father’s occupation					
Service	77	17.62 (3.55)	16.81	18.42	0.169
Unemployed/Business/Other	78	16.92 (2.68)	16.31	17.53	
Mothers occupation					
Service/Other	25	15.70 (2.16)	17.01	18.13	0.006
Homemaker	130	17.57 (3.23)	14.50	15.96	
Daily food intake frequency					
Less than 3 times	87	15.30 (1.59)	14.96	15.64	<0.001
3 to 4 times	33	18.42 (2.19)	17.64	19.19	
More than 4 times	35	21.08 (2.84)	20.10	22.05	

a One-way ANOVA test

**Table 4:** Association of daily food intake frequency with socio-demographic characteristics, BMI percentiles, and hygiene practices

	Daily food intake frequency (%)			P *v*alue	95% Confidence interval	
			
	Less than 3 times	3 to 4 times	More than 4 times		Lower	Upper
Age group						
6 – 9	42 (48.3)	24 (72.7)	15 (42.9)	0.026	0.001	0.051
10 – 12	45 (51.7)	9 (27.3)	20 (57.1)			
Gender						
Male	50 (57.5)	22 (66.7)	18 (51.4)	0.426	0.348	0.504
Female	37 (42.5)	11 (33.3)	17 (48.6)			
Birth order						
1^st^	19 (21.8)	14 (42.4)	7 (20.0)	0.174	0.111	0.234
2^nd^	41 (47.1)	10 (30.3)	16 (45.7)			
>2^nd^	27 (31.0)	9 (27.3)	12 (34.3)			
Categories of BMI percentile						
Underweight	17 (19.5)	-	-	<0.001^a^	<0.001	0.019
Healthy weight	68 (78.2)	21 (63.6)	9 (25.7)			
Overweight/obese	2 (2.3)	12 (36.4)	26 (74.3)			
Washing hand after coming home from outside						
Yes	39 (44.8)	22 (66.7)	34 (97.1)	<0.001^a^	<0.001	0.019
No/irregular	48 (55.2)	11 (33.3)	1 (2.9)			
Brushing teeth per day						
One time	85 (97.7)	30 (90.9)	25 (71.4)	<0.001^a^	<0.001	0.019
Two times	2 (2.3)	3 (9.1)	10 (28.6)			

Total	87 (100)	33 (100)	35 (100)			

a Fisher’s Exact Test
